# Rapid Microsatellite Identification from Illumina Paired-End Genomic Sequencing in Two Birds and a Snake

**DOI:** 10.1371/journal.pone.0030953

**Published:** 2012-02-14

**Authors:** Todd A. Castoe, Alexander W. Poole, A. P. Jason de Koning, Kenneth L. Jones, Diana F. Tomback, Sara J. Oyler-McCance, Jennifer A. Fike, Stacey L. Lance, Jeffrey W. Streicher, Eric N. Smith, David D. Pollock

**Affiliations:** 1 Department of Biochemistry and Molecular Genetics, University of Colorado School of Medicine, Aurora, Colorado, United States of America; 2 Department of Integrative Biology, University of Colorado Denver, Denver, Colorado, United States of America; 3 United States Geological Survey – Fort Collins Science Center, Fort Collins, Colorado, United States of America; 4 University of Georgia, Savannah River Ecology Laboratory, Aiken, South Carolina, United States of America; 5 Department of Biology and Amphibian and Reptile Diversity Research Center, The University of Texas at Arlington, Arlington, Texas, United States of America; Lund University, Sweden

## Abstract

Identification of microsatellites, or simple sequence repeats (SSRs), can be a time-consuming and costly investment requiring enrichment, cloning, and sequencing of candidate loci. Recently, however, high throughput sequencing (with or without prior enrichment for specific SSR loci) has been utilized to identify SSR loci. The direct “Seq-to-SSR” approach has an advantage over enrichment-based strategies in that it does not require *a priori* selection of particular motifs, or prior knowledge of genomic SSR content. It has been more expensive per SSR locus recovered, however, particularly for genomes with few SSR loci, such as bird genomes. The longer but relatively more expensive 454 reads have been preferred over less expensive Illumina reads. Here, we use Illumina paired-end sequence data to identify potentially amplifiable SSR loci (PALs) from a snake (the Burmese python, *Python molurus bivittatus*), and directly compare these results to those from 454 data. We also compare the python results to results from Illumina sequencing of two bird genomes (Gunnison Sage-grouse, *Centrocercus minimus*, and Clark's Nutcracker, *Nucifraga columbiana*), which have considerably fewer SSRs than the python. We show that direct Illumina Seq-to-SSR can identify and characterize thousands of potentially amplifiable SSR loci for as little as $10 per sample – a fraction of the cost of 454 sequencing. Given that Illumina Seq-to-SSR is effective, inexpensive, and reliable even for species such as birds that have few SSR loci, it seems that there are now few situations for which prior hybridization is justifiable.

## Introduction

Constant advances in DNA sequencing technology and lower costs are driving innovation in the life sciences, and are having an especially large impact on the study of ecology, evolution, and population genetics. With these advances, traditional approaches to data generation and marker development require continual re-evaluation. For example, simple sequence repeats (SSRs; also known as microsatellite loci) have long been important in population genetic studies, but the identification of SSRs from non-model species previously required substantial and costly technical effort, and often returned far fewer loci than were required to address most population genetics questions adequately. This effort included creating libraries enriched for SSR loci, cloning, hybridization to detect positive clones, plasmid isolation, and Sanger sequencing. The application of next-generation sequencing approaches has recently made the cost of obtaining SSR loci less expensive and more efficient, allowing researchers to focus technical efforts on obtaining larger sample sizes appropriate to answer the population genetics questions being asked.

Several research groups [1], [2], [3], [4], including our own [5], have developed approaches and software to identify SSR loci from raw 454 sequence reads. These approaches first identify reads containing SSR loci and then identify flanking sequences appropriate for PCR primer sites, avoiding sequences that form secondary structures or are of low complexity. This produces what we call a “potentially amplifiable locus” (PAL). Some of these published approaches included an SSR enrichment step, while others obtained sequence data from an un-selected shotgun genomic library (defined here as the direct “Seq-to-SSR” approach). The long read lengths afforded by 454 sequencing were considered central to this approach because they could identify SSRs and enough flanking sequence on either side for the design of PCR primers. For many species and studies, the number of SSR loci obtained from a small amount of sequencing without enrichment is sufficient [5]. SSRs are rare in the genomes of some species, however, and the prohibitive cost of sufficient 454 sequencing in such cases often necessitates a pre-sequencing SSR enrichment step.

While the per-base cost of 454 sequencing has stayed relatively constant, the cost of obtaining Illumina sequence data has dropped substantially. Illumina sequences now can produce moderately long reads (up to 150 bp with the GAIIx, and 100 bp with the HiSeq) and accommodate paired-end sequencing from both ends of ∼200–600 bp fragments. There have also been massive increases in the number of reads obtained per Illumina sequencing run. To take advantage of these advances, we implemented and tested a new approach, analogous to the previous 454-based method, utilizing Illumina paired-end sequencing to identify PALs (SSR loci and flanking PCR primer sites) without library enrichment or post-sequencing assembly of reads.

We first applied this approach to detect SSR loci from the Burmese python (*Python molurus bivittatus*), which has been shown to have a relatively high genomic frequency of SSR loci [6], [7]. Using libraries prepared from the same python individual, we compared the Illumina results to results using 454 sequencing of an analogous shotgun genomic library. To further demonstrate the utility of using Illumina sequencing for SSR identification, we tested the approach on two bird species (Gunnison Sage-grouse, *Centrocercus minimus*, and Clark's Nutcracker, *Nucifraga columbiana*). We specifically chose to test the approach on birds because, among vertebrates, they have particularly low genomic SSR content [8], [9] and thus can be challenging for shotgun sampling methods. We identified thousands of SSR loci from all samples, but with orders of magnitude better economy using the Illumina-based Seq-to-SSR method.

## Materials and Methods

### Preparation of shotgun libraries

All tissues used in this study were obtained from collaborators, and not collected directly by the authors. Liver tissue (snap-frozen in liquid nitrogen and stored at −80°C) from a captive bred Burmese python was used as a source for genomic DNA (IACUC A08.025, University of Texas Arlington). Total DNA was extracted using standard phenol-chloroform-isoamyl alcohol organic separation, precipitated with ethanol/sodium acetate, washed with 70% ethanol, and resuspended in TE buffer. A total of 10 ug of this DNA was used to make two 454 shotgun libraries, one with FLX shotgun adapters and a second with FLX-Titanium adapters, both prepared using the standard shotgun library preparation protocol and quality control steps (Roche). Data from these two libraries have been previously published [7], and are available on NCBIs Sequence Read Archive (SRA029568), and at www.snakegenomics.org.

An Illumina paired-end (IPE) shotgun library was also prepared from 5 ug of DNA extracted from the same python individual, using a previously published protocol [10] involving fragmentation via nebulization, “Y”-adapter ligation, and agarose gel-based size selection. The resulting paired-end library, including the ligated adapter sequences, had a mean size of approximately 325 bp. This library preparation method used only ‘off-the-shelf’ reagents rather than library preparation kits to reduce the cost to approximately $20 per library.

In addition to the python, total genomic DNA was obtained from two bird species. For the Gunnison Sage-grouse, DNA was extracted from blood obtained from an individual trapped in Gunnison, Colorado (animal protocols approved and conducted by the Colorado Division of Wildlife). DNA was isolated using standard phenol-chloroform-isoamyl alcohol organic separation, precipitated with ethanol/sodium acetate, washed with 70% ethanol, and resuspended in TE buffer. For the Clark's Nutcracker, DNA was extracted from muscle tissue of an individual using the Wizard Genomic DNA Purification Kit (Promega). That individual Nutcracker had been trapped near Logan, Utah and used for behavioural experiments (IACUC number 00-006, Northern Arizona University). Illumina paired-end libraries for both birds were prepared following the same protocol as for the python, and the resulting paired-end libraries had a similar mean size of approximately 325 bp.

Sequencing of the python 454 libraries is described elsewhere [7], but in brief included sequencing on the 454GS platform using either FLX-LR or FLX-XLR Titanium sequencing reagents. About half of the ∼30 million base-pairs (Mbp) obtained came from each of these two sequencing kits, and thus the python 454 data are nearly an equal mixture of FLX-LR and FLX-XLR Titanium sequence reads. Illumina sequencing for the python library was conducted on a GAIIx platform, and sequenced for 114 bp for each of the two paired-end reads. The two bird libraries were sequenced on the GAIIx platform with 120 bp paired-end reads, although the first four nucleotides were multiplex identifiers that were computationally removed, making the effective lengths used for analyses 116 bp per read for both birds.

### Identification of SSR loci

<1?tlsb=-.025w?>A Perl script was written, which we named ***PAL_FINDER_v0.02.03*** *,* to extract reads that contained perfect dinucleotide (2mer), trinucleotide (3mer), tetranucleotide (4mer), pentanucleotide (5mer), and hexanucleotide (6mer) tandem SSRs. Reads were identified as SSRs if they contained simple repeats of at least 12 bp in length for 2–4mers (*e.g.,* 6 tandem repeats for dinucleotides), and at least 3 repeats for 5mers or 6mers. The reads were then sorted by the monomer sequence of the repeat (e.g., TAC or TA repeats) and by the number of tandemly repeated units observed. Non-unique repeat motifs (reverse-complement repeat motifs (*e.g.*, TG and CA) and translated or shifted motifs (*e.g.*, TGG, GTG, and GGT)) were grouped together, so that there were a total of four unique 2mer repeats, 10 unique 3mer repeats, and so on. If multiple SSR loci were discovered in a single read, the locus was considered a compound repeat if the SSR had different motifs; they were considered a broken repeat if the SSR had the same motif. In relatively rare cases in which the same repeated motif occurred at the internal termini of both paired-end sequences, the microsatellite was considered to be a spanning read, and annotated as such.

The program ***PAL_FINDER_v0.02.03*** is operated using a control file that determines parameter settings. The control file can be readily modified by the user to alter criteria for SSR identification. For example, the user can specify which type of reads (454 vs. Illumina) are to be analyzed, and if the program should attempt to design primers or simply count SSR loci. The user can also specify the minimum number of tandem repeats (for each n-mer size class) to be considered, and which n-mer size classes to search for (from 2mers to 6mers).

### Automated design and characterization of PCR amplification primers flanking identified SSR loci

A common motivation for identifying new SSR loci is to use them for scoring allelic length variation. Thus, newly identified SSR loci are typically useful only if primers in the non-SSR flanking regions can be designed and used successfully for PCR amplification. We therefore screened reads with SSR loci for flanking regions with high-quality PCR priming sites. The primer-pair design process was automated to submit large batches of sequences to a local installation of the program Primer3 (version 2.0.0) [11], and was implemented in the Perl program ***PAL_FINDER_v0.02.03*** *,* which is freely available (see below).

For the purpose of selecting primer sites, low complexity and simple repeat sequences were masked from sequences flanking SSR loci using the RepBase v14.01 database (the “simple.txt” library) [12]. We used the following criteria for primer design: 1) GC content greater than 30%; 2) melting temperatures of 58–65°C with a maximum 2°C difference between paired primers; 3) the last two 3′ nucleotides were G or C (a GC “clamp”); 4) maximum poly-N of four nucleotides. All other parameters were set to Primer3 default values. If all criteria were met, a single primer-pair was chosen based on the highest score assigned by Primer3 [11], and based on finding primers that will amplify the maximum number of repeats in each read or read pair. The control file for the ***PAL_FINDER_v0.02.03*** program contains a large number of parameter settings that direct the primer design criteria of Primer 3. These include ranges and optimal values for primer length, melting temperature, and secondary structure. The user can readily modify these parameter settings in the ***PAL_FINDER*** control file.

A concern in identifying PALs is the copy number of the primer sequence in the genome. We addressed this by estimating the number of observed occurrences of identified primer sequences in the sequence set analyzed. Specifically, ***PAL_FINDER*** uses the raw set of reads as a reference and counts the copy number of forward and reverse primers in this library. A further consideration is that while the forward and reverse primer sequences may have multiple copies in the genome, they can still produce a single distinct band for scoring SSRs if they occur close to one another only once or a few times. In other words, even if primer sequences are somewhat frequent in the genome sample, they may only rarely occur in close proximity (and thus produce a PCR product). To evaluate this, we counted how often each PAL primer pair co-occurred in a set of paired reads in our library of reads for each species. Thus, PALs can be further screened based on the copy number of primers and primer pairs, with the lowest frequencies indicating primers and pairs most likely to amplify a single locus. All these attributes of PAL primers are annotated for each locus in the output of ***PAL_FINDER*** *.* These attributes of primer copy number, together with their sequences, and the detailed detection of SSRs per locus are output in a combined tab-delimited. This allows the output of SSR loci with flanking primers to be sorted and filtered by a number of criteria that might interest researchers.

## Results

### Raw data and subsets used for demonstration

The genome size of the Burmese python is unknown, but a related python species (*P. reticulatus*) has been estimated to be 1.44 Gbp [13]. The genome sizes for the two birds used in this study are also unknown, although bird genomes that have been surveyed average 1.38 Gbp [14]. Thus, for rough comparisons of SSR loci identification and sequence sampling, the python and the two birds in this study can be considered to have approximately similar genome sizes. For the purposes of comparing the success rates of SSR identification across species using the Illumina-based Seq-to-SSR method, we chose to use datasets including 5 million paired-end reads (equivalent to 5 million×2 reads; 5 M hereafter) per species. This number was chosen because it was slightly under 1× nucleotide-level coverage (∼1.15 Gbp) of the genomes of these species.

For detailed comparisons of performance of 454 versus Illumina-based sequencing, we focused analyses on the python, for which we had both types of data from the same individual. In a previous study [7], we had collected 28.5 Mbp from 118,973 reads from shotgun genomic libraries using the 454 platform (available at www.snakegenomics.org, and NCBI's Sequence Read Archive, accession SRA029568), and we use these data to evaluate the performance of the 454 reads in the Seq-to-SSR approach. For direct comparisons with the 454 data, we subsampled the python Illumina data to include the same number of reads as the 454 data: 118,973 paired-end reads. Hereafter, we refer to these 454 and Illumina paired-end datasets as “454” and “IPE-119K”, as in Table 1.

**Table 1:** Summary of microsatellite identification from various python and bird genome sequence sets.

	Burmese Python			Gunnison Sage-grouse	Clark's Nutcracker
Sample set	454	IPE-119K	IPE-5M	IPE-5M	IPE-5M
Millions of reads	0.119	0.119	5.000	5.000	5.000
Megabases of sequence	28.5	27.1	1,140.0	1,160.0	1,160.0
Reads containing one or more microsatellites	11,027	11,073	470,333	228,243	179,663
Total individual microsatellite loci	13,142	12,833	546,956	247,714	195,176
Compound loci	1,314	973	41,726	8,756	4,528
Mirosatellite reads per megabase of sequence	386.9	408.6	412.9	196.8	154.9
Discrete PALs	5,474	4,129	174,370	74,606	72,125
Discrete PAL rate	0.496	0.373	0.371	0.327	0.401

### Comparison of 454 and Illumina-based Seq-to-SSR with the python

The 454 and IPE-119K python data contained the same number of reads and similar amounts of sequence (28.5 Mbp and 27.1 Mbp, respectively), and were comparable in their ability to identify SSR loci and flanking primers (Table 1). Just over 11,000 sequences containing SSR loci were identified in each data set (454: 11,027, IPE-119k: 11,073), and the total number of SSR loci identified in each were similar (454: 13,142, IPE-119k: 12,833), being slightly higher than the number of SSR-containing reads because some reads contained multiple SSRs. Thus, SSR loci were identified from between 9.23% (454) and 9.37% (IPE) of all python reads from both platforms. We identified 5,474 PALs from the 454 data and 4,129 PALs from the IPE-119k data (Table 1), with about one quarter of each of these sets containing multiple (compound) SSRs. Thus, fairly similar proportions of PALs from among the distinct SSR-containing loci were identified in both data sets (49.6% from the 454 data and 37.3% from the IPE-119K data), with about 12% greater PAL identification success from the 454 reads that contained SSRs.

**Figure 1:**
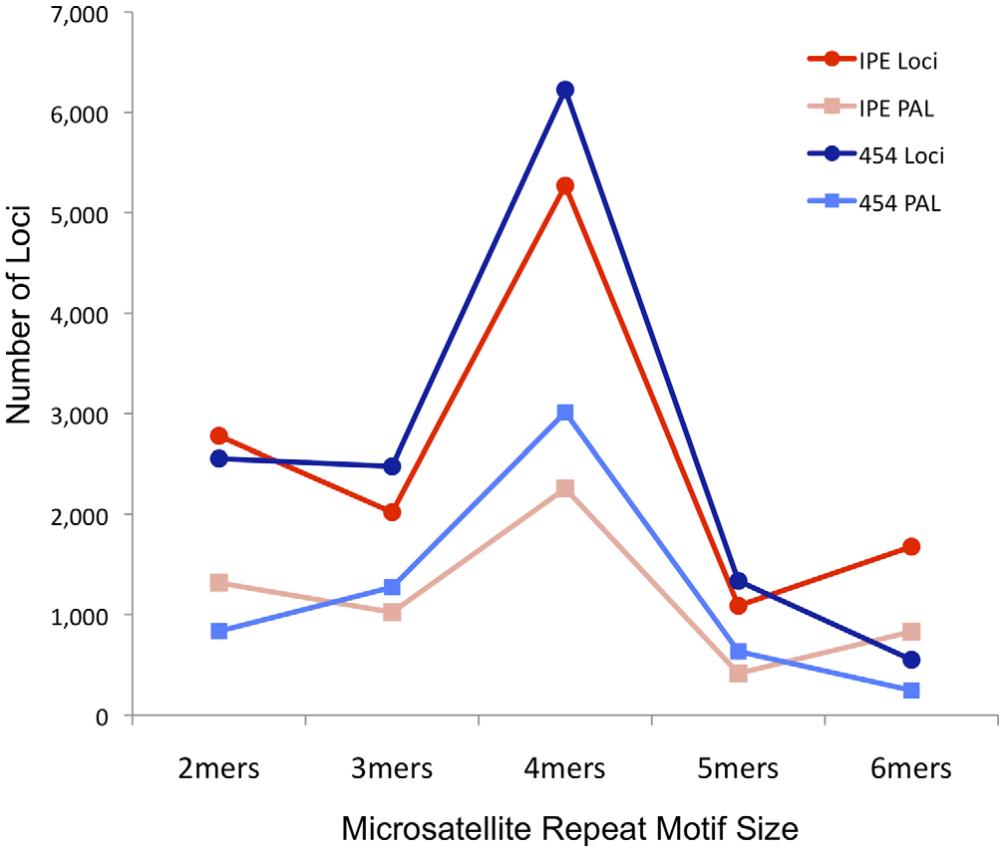
Comparison of identification of microsatellite loci and ‘potentially amplifiable microsatellite loci’ (PAL) using Illumina long (114 bp) paired-end reads versus 454 reads. Comparison based on the same number of reads for each platform (118,973 reads) sampled from Burmese python shotgun genomic libraries.

To determine whether there were differences between the techniques based on SSR structure, we further examined SSR locus and PAL identification by repeat motif monomer size (Fig. 1). More SSRs and PALs are identified from analysis of IPE reads (versus 454) for the shortest and longest repeat motifs (2mers and 6mers), whereas the opposite is true of the middle-sized repeats, such as the 4mers (Fig. 1, Table 2). These differences in identification success between 454 and IPE reads are highly significant for each of the SSR n-mer classes (P<0.001, based on *G*-tests). Their basis is uncertain, but may be due to differences in sequencing performance in highly repetitive regions.

**Table 2:** Comparison of microsatellite and PAL identification from Illumia paired-end reads versus 454 reads for the Burmese Python, broken down by microsatellite repeat motif length.

	2mers	3mers	4mers	5mers	6mers
IPE-119K					
Loci	2,778	2,020	5,270	1,088	1,677
PAL	1,317	1,023	2,256	415	830
Percent PAL	47.41%	50.64%	42.81%	38.14%	49.49%
454					
Loci	2,554	2,476	6,226	1,336	550
PAL	835	1,273	3,012	633	245
Percent PAL	32.69%	51.41%	48.38%	47.38%	44.55%

### Comparison of PAL recovery for the python versus bird genomes with Illumina Seq-to-SSR

We compared the effectiveness of Illumina Seq-to-SSR in two birds and the python using five million IPE reads each (henceforth, we refer to these as IPE-5M data sets). As expected based on previous information on the relative abundance of SSR loci in bird and snake genomes [6], [7], [8], [9], [15], [16], [17], we identified about twice as many SSR loci (Table 1) in the python (546,956) as in the Gunnison Sage-grouse (247,714) or Clark's Nutcracker (195,176) IPE-5M samples. There was some difference among the three species in the rate of PAL identification, with the highest rate in the nutcracker (40.1%), an intermediate rate in the python (37.1%), and the lowest rate in the grouse (32.7%; Table 1).

**Figure 2:**
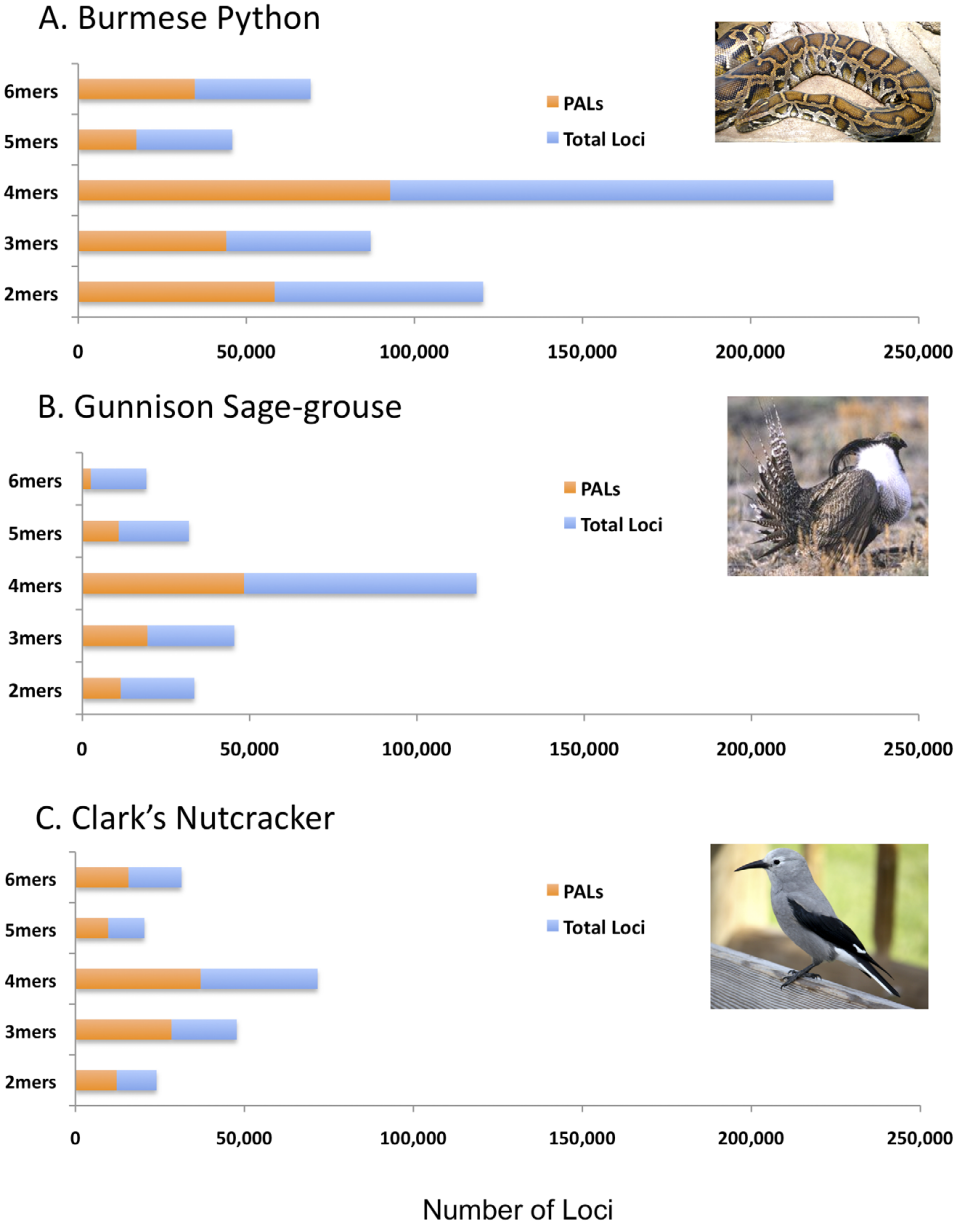
Comparison of identification of microsatellite loci and ‘potentially amplifiable microsatellite loci’ (PAL) among bird and python samples. Analysis based on using five million Illumina long (114–116 bp) paired-end reads from a shotgun genomic library for each species.

**Figure 3:**
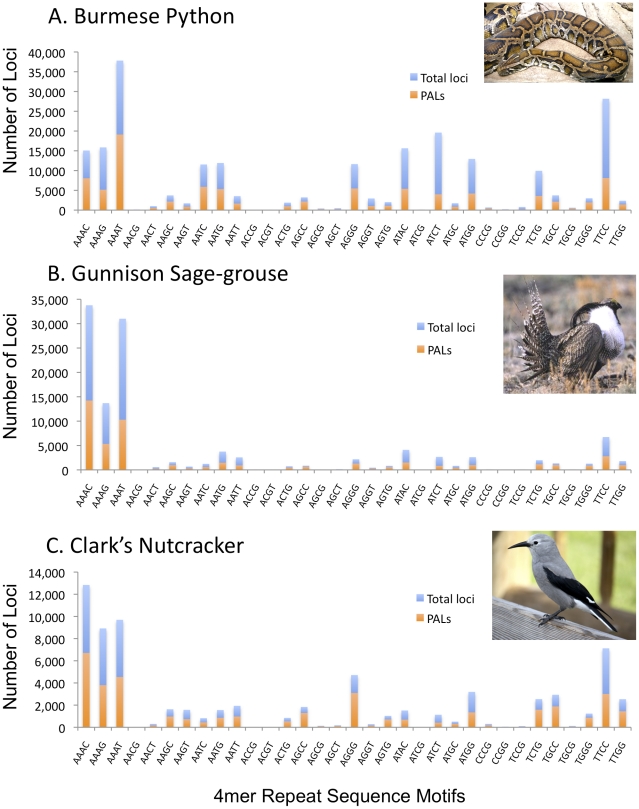
Comparison of microsatellite loci and ‘potentially amplifiable microsatellite loci’ (PAL) identification for 4mer repeat motifs in birds and the python. Results based on five million Illumina long (114–116 bp) paired-end reads per species.

The three genomes have notably different frequencies of SSRs of different repeat motif lengths (*e.g.,* 3mers, 4mers). The 4mers are most frequent in all three genomes, and particularly abundant in the python (Fig. 2). The python genome also had particularly high counts of 6mer repeats compared to the two bird genomes (Fig. 2). As an example of differences in motif composition, we compared motifs for 4mers, which are desirable for scoring amplified loci based on size because they are abundant, generally variable, and more easily scored than shorter motifs (Fig. 3). Some 4mer motifs (*e.g.*, AAAC, AAAG, AAAT) have similar frequencies among the birds and the python, but many others differ substantially in frequency between the python and birds, and even between the two bird species (*e.g.*, TTCC, ATCT, ATGG; Fig. 3). These differences highlight the strength of the Seq-to-SSR approach in discovery of SSR loci without requiring *a priori* targeting of particular motifs, as in enrichment-based approaches, because it would be difficult predict these within-class differences in motif frequencies ahead of time.

### Copy number of primer sites flanking SSRs

To utilize the identified SSR loci in PCR-based size scoring of alleles, it is important to determine which loci will successfully produce PCR amplification products. A major concern is that primers designed in the flanking regions around these loci only amplify a single locus. Therefore, as part of our annotation scheme we applied three overlapping degrees of stringency (criteria) for filtering PALs, based on how their primer sequences (or their reverse complements) were repeated in the entire set of reads. The most stringent criterion was to take the product of the counts of the forward and reverse primers in all reads, where a product of “1” would mean that both the forward and the reverse primers were each only observed once in the entire read dataset. (Note that the designation of “forward” and “reverse” primers here is arbitrary, depending on the direction in which they happened to be read). The next stringency level was to take the minimum number of times either primer in a PAL occurred in the entire set of reads. With this filter set to “1”, for example, at least one of the two PCR primers chosen should be unique to the SSR locus targeted, and therefore lead to successful specific amplification. The least stringent criterion depended on the number of times that a pair of PAL primers was observed together, in the correct orientation, in paired reads. This is a direct estimate of how often they might occur in close enough proximity in the genome to produce amplifiable PCR products, but is the least stringent criterion because the amount of sequencing may be insufficient to detect repeated pairs. It is also possible that primer pairs might be amplifiable but are further apart than the lengths of the paired-end library, and are thus not detected.

**Figure 4:**
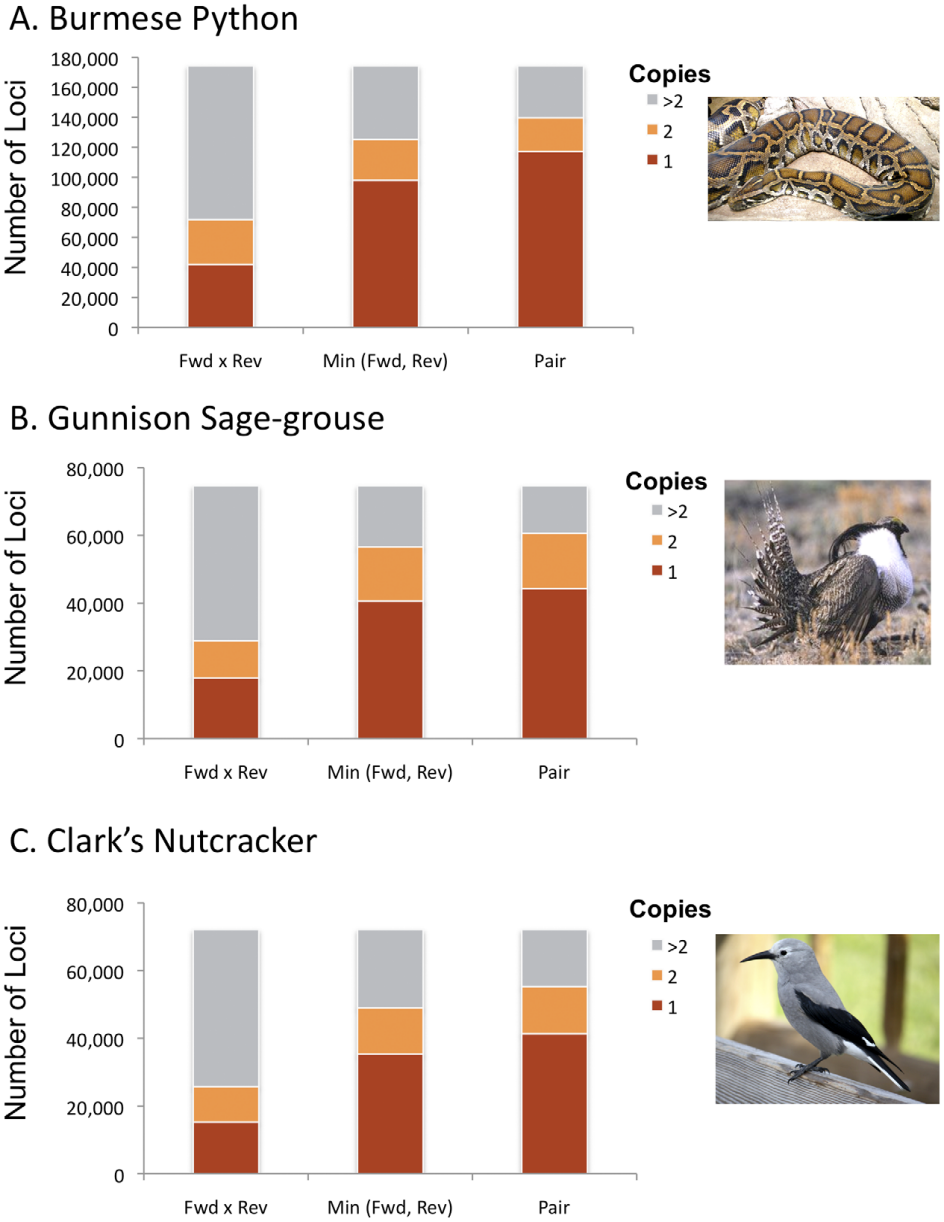
Empirically estimated copy numbers of identified flanking primer sequences for potentially amplifiable microsatellite loci (PALS) for each species. The number of times a primer sequence or primer pair was observed in all data (from five million reads per species) was counted per species to approximate their genomic frequencies. Here the “Min (Fwd, Rev)” represents the minimum copy number of the forward and reverse primers observed in the data. The product of the independent frequencies of the forward and reverse primer sequences (per locus) is also shown (“Fwd×Rev Primer”), as is the frequency that each primer pair was observed together in a set of paired reads (“Primer Pair”).

To decide what the cutoff numbers for each of these stringency criteria should be, we divided the numbers in each stringency criterion into classes based on 1, 2, or >2 observations. The proportions in each category for each stringency criterion were strikingly similar across the three species (Fig. 4), with the proportion of filtered PALs for any given stringency/cutoff combination ranging from about 20% (only one copy of both primers observed) to about 80% (only one or two copies of the primer pair observed in the same orientation in paired reads). By even the most stringent criteria, and for the bird with the fewest SSRs, there were still 15,269 stringently filtered PALs (i.e., for the nutcracker with both PAL primers occurring only once). Thus, filtering potential target PALs based on stringent primer copy number requirements still results in tens of thousands of high-quality loci.

### Yield of ultra high-quality amplifiable loci (Best PALs)

**Figure 5:**
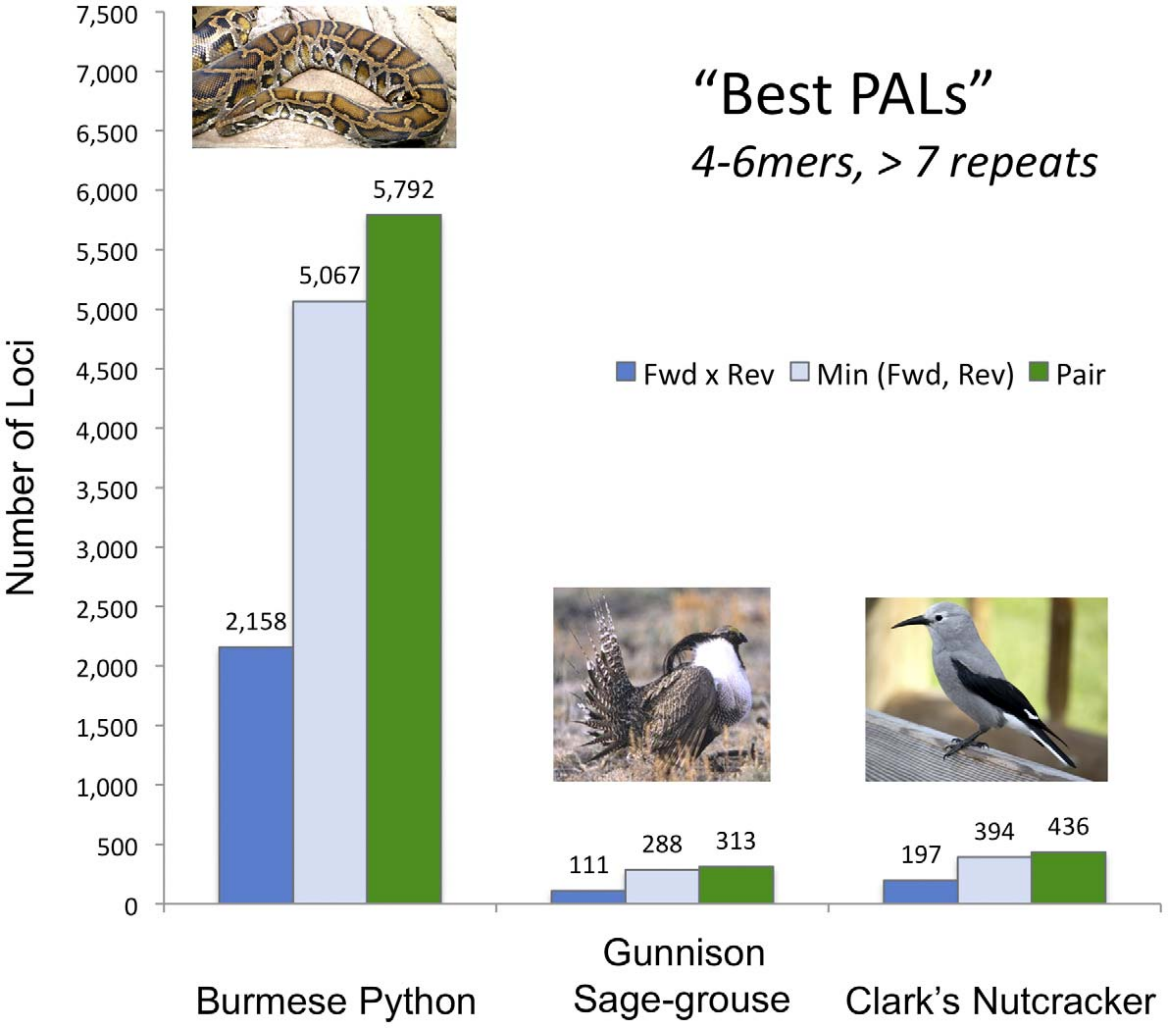
Highly stringent selection of choice microsatellite targets. Microsatellite loci were selected from the 5 million read datasets for each species that fit specific criteria for repeat monomer length (4–6), number of repeats observed (>7), and primer copy number in the observed data (variations shown above).

To further convey the practical return of extremely high quality SSR loci that might be expected from sampling five million Illumina reads, we considered data that were selected for having both long repeat units (4-, 5-, and 6mers, which are more easily scored) and longer repeat stretches (more than 7 observed repeat units, which are more likely to be highly variable in population samples). We refer to this highly selective set of loci as “Best PALs”. We note that while these particular criteria are somewhat arbitrary, these criteria are readily selectable using the control file of the program ***PAL_FINDER_v0.02.03*** *,* and thus readily tuned. We then considered the same three stringency criteria as before (Figure 4), each with a cutoff of 1. In the python, the numbers of such PALs returned ranged from ∼2,100 for the most stringent criterion to ∼5,800 for the least stringent (Fig. 5). In birds, the numbers for the same criteria were, respectively, ∼100–200 and ∼300–450 (Fig. 5). Thus, even though there are far fewer usable SSRs in birds compared to other vertebrates, the massive read numbers offered by Illumina Seq-to-SSR still provide sufficient numbers of loci, even with extremely stringent criteria, for robust population genetic analyses.

### Availability of software and SSR loci identified

Supplementary data are available from the journal's website, and at the lead and corresponding authors' web sites (www.EvolutionaryGenomics.com and www.snakegenomics.org). The Perl script (***PAL_FINDER_v0.02.03***) used to identify and analyze SSR loci is also freely available at http://sourceforge.net/projects/palfinder/and at the authors' web sites. An accessory script for preparing multiplexed IPE data for input into *PAL_FINDER* is available at the authors' websites. Identified sets of SSR loci, together with statistics for each locus, and primer sequences for PALs, are provided online as tab-delimited files for each of the three species analyzed, based on the full 5 million read datasets (Datasets S1, S2, S3). For the python, PAL_FINDER output files based on analysis of the 454 and matched IPE-119 data are provided as supplementary files (Datasets S4, S5).

## Discussion

Our results suggest that Illumina paired-end sequencing is capable of identifying massive numbers of potentially PCR-amplifiable SSR loci with tremendous economy. We find that on a read-by-read basis, Illumina paired-end sequences are nearly as effective as 454 sequence reads for identification of PALs. The levels of PAL recovery from Illumina sequencing are high enough that a fraction of a flow cell (lane) is sufficient to identify tens of thousands of PALs, even in taxa such as birds that have low genomic SSR densities. Thus, with Illumina sequencing, there seems to be little justification for performing an intermediate step of hybridization (targeting specific SSR motifs) prior to sequencing [18], rather than the direct Seq-to-SSR described here.

Currently, the GAIIx is capable of producing ∼30 million reads per flow cell lane (1/8 of a flow cell), and the HiSeq is capable of producing ∼180 million reads per lane, with read lengths of up to 150 bp (GAIIx) and 100 bp (HiSeq) per read, for approximately $2500. In contrast, for a similar price the 454 platform would be expected to deliver approximately 300,000 reads (from a 1/4 70×75 mm picotiter plate), or 100× fewer than the GAIIx. Although the HiSeq platform offers 6-fold greater economy per paired read, the GAIIx platform offers longer read capability (to 150, versus 100 bp), and substantially more accurate base calling at lengths >50 bp. Given that ∼$500 of GAIIx sequencing of the python yielded over a half million identified SSR loci, and ∼175,000 PALs, it seems that the cost of the GAIIx is already sufficiently low that there is no great benefit to using HiSeq and its somewhat shorter and less accurate reads.

As an example of the extreme economy of the method, the cost to sequence the IPE-119K dataset would have been ∼$10 on the GAIIx. Thus, for the python, 1/250 of a GAIIx lane yielded 4,129 PALs. Also, our shotgun library preparations utilized all independently purchased (“off the shelf”) reagents, rather than kits, and cost approximately $20 per sample. Considering that our results demonstrate similar performance for SSR locus and PAL identification on a read-by-read basis for 454 and IPE data, and a two to three order of magnitude difference in cost favouring IPE sequencing, IPE-based SSR identification by Seq-to-SSR is likely the preferred approach.

The major benefit of the IPE Seq-to-SSR approach for evolution, population genetics, and linkage mapping studies is that it quickly, reliably, and inexpensively delivers an unbiased genome-wide characterization of SSR loci along with PALs and their primers. It also produces a rich dataset of randomly sampled sequences that can be used for other purposes, such as studying transposable element content, mitochondrial genomes, or other highly repeated DNA segments. Furthermore, unlike other methods, the Seq-to-SSR approach provides information on the possible repetitive nature of potential primers that no other approach provides. While other groups have used IPE sequencing to identify SSR loci from genomic libraries after enriching for SSRs [18], the economy of IPE sequencing argues strongly against the need for such enrichment. One slight disadvantage of IPE versus 454 sequencing is, however, that 454 reads will often count the exact number of SSRs, whereas the exact number of repeats may be unknown for many IPE loci [18]. This is because the IPE library insert size is typically larger than the combination of the two paired read lengths (as in our case), and therefore, SSR loci may extend into the intervening portion of the insert sequence that is not covered by either read. It is also notable that the total read length of both 454 and IPE data limits the measurement of the total length of SSRs, such that the length of SSRs exceeding the read length or extending outside the boundaries of reads will be underestimated. Despite this, loci can still be sorted and targeted for further work based on the observed number of repeats in the IPE paired reads, which represents a lower bound on the absolute number of repeats.

Given the large number of loci identified, the Seq-to-SSR approach allows great flexibility to preferentially target loci with favourable characteristics. For example, longer SSRs [19] are generally known to exhibit greater allelic variability, as are perfect (versus imperfect or compound) SSRs [20]. Other characteristics of a locus, such as the copy number of designed flanking primers and length of targeted amplicon, determined by the Seq-to-SSR approach, provide further information for choosing loci most likely to amplify successfully.

Here, we provided one example of extremely stringent filtering of PALs to identify a set of “Best PALs” that have many of the above mentioned characteristics, as well as having longer repeat motifs (4–6mers). These strict criteria yielded hundreds of Best PALs in the birds and thousands in the python. These and many other features of loci are either adjustable parameters in our program ***PAL_FINDER***, or are part of the output annotation for each locus and can thus be used to sort and filter sets of SSRs. To empirically demonstrate the effectiveness of the approach, we applied these same “Best PALs” filters to similar IPE data from a plant (*Mimulus ringens*), and empirically tested 48 highly stringent primer sets on four individuals. In the first attempt, 22 of these loci produced clearly distinguishable amplification products, and 21 were polymorphic. Another 9 were probably good polymorphic loci but require further PCR optimization. Thus, by sampling microsatellite loci on essentially a genome-scale, the IPE Seq-to-SSR approach provides excellent flexibility for researchers to choose microsatellite loci with a suite of favourable characteristics for their needs.

Our approach and software for SSR loci primer identification should also be useful to rapidly characterize genomic SSR landscapes for comparative purposes. We used the earlier 454-specific version of this software to identify differences in the genomic SSR content among species of snakes. This led to the discovery that these differences were due to SSR-seeding by a particular family of transposable elements [7]. In the current study, our analysis showcases the major differences between bird and snake SSR content, and newly demonstrates substantial differences in SSR content between the two bird genomes (e.g., Fig. 3).

## Supporting Information

Dataset S1 **Potentially amplifiable loci output - Burmese python,** ***Python molurus bivittatus*** **(5 million read IPE dataset).** This is a tab-delimited text file that can be readily imported into a spreadsheet.
(TXT)

Dataset S2 **Potentially amplifiable loci output - Gunnison Sage-grouse,** ***Centrocercus minimus*** **(5 million read IPE dataset).** This is a tab-delimited text file that can be readily imported into a spreadsheet.
(TXT)

Dataset S3 **Potentially amplifiable loci output - Clark's Nutcracker,** ***Nucifraga columbiana*** **(5 million read IPE dataset).** This is a tab-delimited text file that can be readily imported into a spreadsheet.
(TXT)

Dataset S4 **Potentially amplifiable loci output - Burmese python,** ***Python molurus bivittatus*** **(119k IPE dataset).** This is a tab-delimited text file that can be readily imported into a spreadsheet.
(TXT)

Dataset S5 **Potentially amplifiable loci output - Burmese python,** ***Python molurus bivittatus*** **(454 dataset).** This is a tab-delimited text file that can be readily imported into a spreadsheet.
(TXT)
