# Correction: Rapid Microsatellite Identification from Illumina Paired-End Genomic Sequencing in Two Birds and a Snake

**DOI:** 10.1371/journal.pone.0136465

**Published:** 2015-08-20

**Authors:** Todd A. Castoe, Alexander W. Poole, A. P. Jason de Koning, Kenneth L. Jones, Diana F. Tomback, Sara J. Oyler-McCance, Jennifer A. Fike, Stacey L. Lance, Jeffrey W. Streicher, Eric N. Smith, David D. Pollock

In the Materials and Methods, an Institutional Animal Care And Use Committee (IACUC) protocol number was incorrectly cited. The authors wish to correct the record by reporting that there was no University of Texas at Arlington (UTA) IACUC protocol number for this research because the research was not conducted at UTA, with the exception of the dissection of the python, which took place without UTA IACUC approval. The corrected text and additional details and clarification are provided below for the tissue sample from the Burmese python, as well as the samples from two birds, the Gunnison Sage-grouse and Clark's Nutcracker.

"All specimens used in this study were obtained from collaborators and not collected by the authors. There was no University of Texas at Arlington (UTA) IACUC protocol number for this research as the research was not conducted at UTA, with the exception of the dissection of the python, which took place without UTA IACUC approval. A single *Python molurus bivittatus* was obtained by the UTA Amphibian and Reptile Diversity Research Center from a commercial breeder in August of 2007, immediately euthanized using a chloretone solution, and tissues preserved following animal protocols outlined in the Guidelines for Use of Live Amphibians and Reptiles in Field Research, established jointly by the American Society for Ichthyologists and Herpetologists, Herpetologist's League, and the Society for the Study of Amphibians and Reptiles (http://www.aaalac.org/accreditation/Guidelines_for_Use_of_Live_Amphibians_and_Reptiles.pdf). Liver tissue from the python (snap-frozen in liquid nitrogen and stored at -80°C) was used as a source for genomic DNA.

The Gunnison Sage-grouse was trapped and blood was sampled by personnel from the Colorado Division of Wildlife. The trapping method and blood sampling was approved through their Animal Care and Use Committee; however, at the time, no permit was required to trap and sample the bird (the bird was still being actively hunted at the time when the sample was taken). There was no number issued in terms of an IACUC.

The tissue from the Clark's Nutcracker was provided from a dead carcass of a bird that died of natural causes in the lab of Russell Balda at Northern Arizona University after more than a decade as an experimental bird for memory studies. The bird was originally caught in Logan Canyon in the 1990s by Stephen Vander Wall (University of Nevada Reno), under the IACUC protocol number 00–006 Russell Benford, Ph.D., Department of Biological Sciences, Northern Arizona University.
